# Everyday Conflict in Families at Risk for Violence Exposure: Examining Unique, Bidirectional Associations with Children’s Anxious- and Withdrawn-Depressed Symptoms

**DOI:** 10.1007/s10802-022-00966-6

**Published:** 2022-11-04

**Authors:** Nicholas M. Morelli, Kajung Hong, Jackelyne Garcia, Xavier Elzie, Andrew Alvarez, Miguel T. Villodas

**Affiliations:** 1 San Diego State University/University of California, San Diego Joint Doctoral Program in Clinical Psychology, San Diego, US; 2 Department of Psychology, San Diego State University, 6363 Alvarado Ct., Suite 250, 92120 San Diego, CA US

**Keywords:** Family conflict, internalizing, bidirectional, childhood

## Abstract

Exposure to family conflict during childhood increases risk for the development of anxiety and mood problems, though the potential for bidirectionality in this association remains unknown. It is also unclear whether nonviolent family conflict is related to children’s anxious- and withdrawn-depressive symptoms within high-risk family contexts, independent of more severe events such as children’s exposure to violent victimization. Participants included 1,281 children and their caregivers identified as being at high risk for family violence, interviewed prospectively at ages 6, 8, and 10 about family conflict, children’s anxious- and withdrawn-depressive behaviors, and children’s victimization experiences. After controlling for cumulative victimization prior to and between each time point, significant cross-lagged, bidirectional associations were identified between family conflict and children’s anxious- and withdrawn-depressive symptoms across ages 6 and 8, but not across ages 8 and 10. Invariance testing revealed no differences in model fit between boys and girls and minimal differences depending on whether families were reported for maltreatment prior to recruitment. Findings are partially supportive of a conceptualization of the family-child relationship that is reciprocal, while highlighting the role of daily negative familial interactions, over and above experiences of victimization, in predicting anxious- and withdrawn-depressed symptoms in early and middle childhood.

**Supplementary Information:**

The online version contains supplementary material available at 10.1007/s10802-022-00966-6.

The family system represents the most proximal social environment for children. Frequent and/or high levels of conflict within the family, including arguing, competition, and criticism between family members, is associated with childhood depression and anxiety (Choi et al., 2020; Davis & Epkins, 2009; Rabinowitz et al., 2016a). Family conflict is also a risk factor for more severe forms of dysfunction, such as children’s victimization by violence (Silber et al., 1993; Stith et al., 2009). The co-occurrence of family conflict and violent victimization makes it difficult to disentangle their unique impact on children’s socioemotional development. Further, the empirical literature has typically conceptualized the family system as predicting children’s socioemotional development unidirectionally (Pardini, 2008; Zvara et al., 2018), despite prominent theoretical models (e.g., developmental psychopathology) emphasizing the reciprocal, interactive nature of children and their environment (Cicchetti, 2016). In sum, it remains unclear whether the association between family conflict and child anxious and depressive symptoms (1) functions bidirectionally and (2) persists above and beyond children’s exposure to violent victimization. The present study attempts to address these gaps by prospectively examining bidirectional and transactional associations between family conflict and children’s caregiver-reported internalizing problems across three timepoints during middle childhood, above and beyond children’s cumulative victimization experiences. The invariance of these models was then tested in boy and girls and in youth who did or did not have child maltreatment reports prior to study recruitment. The families included in this study represent a diverse, socioeconomically-disadvantaged, and high-risk population based on previous involvement in child welfare services and other demographic risk factors (e.g., poverty, single-parenthood).

## Family Conflict and Children’s Anxious and Depressive Symptoms

Theories of family systems (e.g., “holism”) argue that the family unit represents more than an additive aggregation of functioning within each family subsystem. According to this conceptualization of the family unit, examining the impact of any one subsystem within the family, such as the interparental or parent-child relationship, is insufficient for understanding the full impact of the family environment on children’s development (Cox et al., 2001). The concept of family conflict was developed in line with this principle to represent the maladaptive ways in which families communicate and solve problems together. As it is defined here, family conflict refers to nonviolent patterns of arguing, fighting, competition, favoritism, rejection, criticism and blame within the family system as a *whole* (Beavers et al., 1985, 1990). In this way, family conflict includes but extends beyond more specific, dyadic forms of negative interpersonal interactions (e.g., parent-child or interparental conflict). Family conflict differs qualitatively from more severe forms of conflict, such as physical abuse, intimate partner violence, or exposure to community violence, in that it represents lower-level, nonviolent forms of negative interactions likely to occur on a day-to-day basis.

Family conflict is linked with a wide range of internalizing problems in children, including depression, anxiety, global distress, and suicide (Anyan & Hjemdal, 2018; Au et al., 2009; Jozefiak & Wallander, 2016; Kelly et al., 2016; Negy & Snyder, 2006; Rabinowitz et al., 2016b; Rapp et al., 2017). Consistent with the idea of holism, these associations are often observed over and above the effects of individual- and dyadic-level characteristics such as parenting styles, parent history of psychopathology, children’s negative cognitive style, and parents’ marital quality (Buehler et al., 2007; Marmorstein & Iacono, 2004; McHale & Rasmussen, 1998; Rapp et al., 2017). While theorized underlying mechanisms vary, the chronic interpersonal stress, social defeat, and criticism associated with family conflict are likely to promote negative self-schemas, lower expectations for social rewards, and teach maladaptive strategies for coping with negative affect (Roubinov & Luecken, 2013; Sameroff, 1995).

## Impact of Family Conflict Versus Violent Victimization

All families will experience conflict to some degree. For some, however, conflict that is frequent and severe may pose an additional threat to children by increasing risk for more dangerous forms of family dysfunction in the household, such as the use of violence (Silber et al., 1993; Stith et al., 2009). For example, conflict, low cohesion, and poor problem solving within the family unit are all associated with parents’ increased risk of using of physically abusive discipline strategies (Schaeffer et al., 2005; Thurman & Kidman, 2011). When nonviolent family conflict and violent victimization co-occur within a family, a crucial question arises regarding their unique impact on children’s socioemotional development. Nonviolent family conflict, though less severe than violent victimization, still represents a frequent and potent interpersonal stressor for children, chronically reactivating neurobiological and psychophysiological regulatory processes and promoting negative cognitive-behavioral coping strategies (Harold & Sellers, 2018). Family conflict, as it is defined here, also tends to occur more frequently than violence or abuse (Kim & Drake, 2019). It is plausible that everyday family conflict influences the development of children’s anxious and depressive symptoms above and beyond the effects of more severe, but less frequent, violent events; however, to date, no empirical studies have confirmed this. Understanding the impact of chronic, lower-level stressors, even in the presence of more stressful victimization experiences, would inform the development of more effective and targeted family-based interventions in high-risk families. Delineating the unique impact of family conflict on children’s development is critical.

## Bidirectional Family-Child Effects

Developmental theorists have long argued that children’s socioemotional outcomes are the product of countless transactional interactions with their environment (Cicchetti, 2016), yet empirical studies often continue to frame family-child effects as unidirectional. As a result, much work has been done to establish the association between family conflict and children’s socioemotional problems (Choi et al., 2020; Davis & Epkins, 2009; Rabinowitz, Drabick, et al., 2016), but few studies have explored the reverse association. It is conceivable that children’s internalizing problems, through no fault of their own, could exacerbate family conflict. This may happen directly via maladaptive responses by family members. Children’s withdrawn and disengaged coping strategies, for example, have been associated with increased stress from family members (Roubinov & Luecken, 2013; Rubin & Mills, 1992). Anxious behaviors such as reassurance seeking and school refusal can also lead to frustration by parents and problems at school, increasing conflict within the household (Joiner & Coyne, 1999). Children’s emotion dysregulation, a transdiagnostic correlate of all internalizing problems, has been prospectively linked to increases in parenting stress (Williford et al., 2007). Children’s internalizing problems may also exacerbate conflict in more indirect ways, for example, through the family’s involvement with mental health services, which places an additional financial and temporal burden on family members. The potential for children’s internalizing problems to worsen family conflict is especially relevant in families involved in child welfare services, affected by poverty, or lacking familial and community support. These families, to a greater degree than others, may lack the economic, social, and psychological resources to manage and accommodate a child’s mental health problems (Mazur & Mickle, 2018).

A sizable body of work has examined bidirectional processes in the context of narrowly-defined dyadic interactions or parent-level factors, such as parent-child conflict, aggressive parenting, or parental mental health. This work has supported the idea that child behavior problems and negative parenting practices can exacerbate one another over time (Akcinar & Baydar, 2016; Pardini, 2008; Wiggins et al., 2014). For example, in a longitudinal study of low-income parent-child dyads, negative parenting practices (i.e., poor support and structure) at child age 7.5 were associated with an increase in internalizing symptoms at age 10.5, which was in turn associated with an increase in negative parenting practices three years later at age 13.5 (Serbin et al., 2015). Utilizing a large prospective pregnancy cohort, Hentges et al., (2021) found that postpartum depression was associated with an increase in children’s internalizing problems at 3 years old, which in turn was associated with an increase in hostile parenting two years later at age 5. Similar findings involving interparental conflict have been documented. In a study of (*N* = 660) children ages 8–11 who had experienced separation from parents, children’s internalizing problems (but not externalizing problems) were associated with an increase in interparental conflict 6 months later, which was in turn associated with an increase in children’s internalizing and externalizing problems at a third time point 6 months thereafter (Wang et al., 2022).

These studies provide broad evidence for bidirectional parent-child effects involving children’s internalizing problems. Still, little work has explored this topic in the context of the broader family unit. One such study by Kelly et al., (2016) identified bidirectional associations between family conflict and depression across early adolescence, including a transactional process linking age 13 depressive symptoms with age 15 depressive symptoms through age 14 family conflict. No studies of which we are aware have modeled bidirectional patterns of family conflict and internalizing psychopathology prior to adolescence. This is important given that childhood represents a period of heightened vulnerability to influence from the family environment, before peer relationships and social acceptance compete as the primary determinants of socioemotional functioning (Brown & Larson, 2009).

## Present Study

**Fig. 1:**
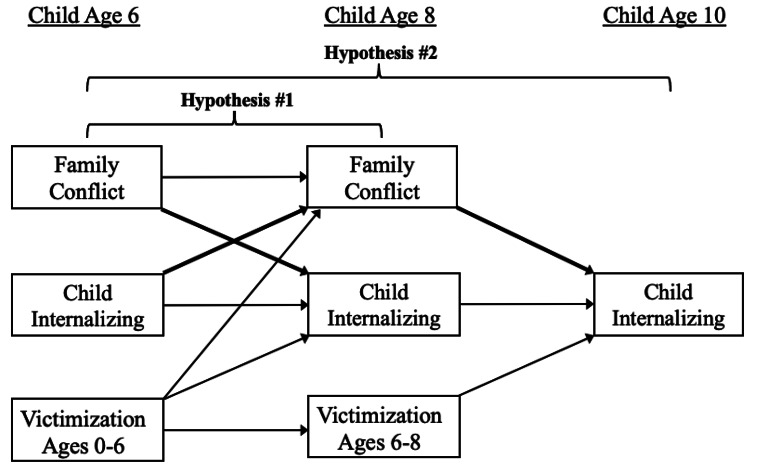
Conceptual model depicting cross-lagged and autoregressive paths between family conflict and child internalizing problems across ages 6 and 8 (Hypothesis #1) and a transactional process indicated by paths from age 6 child internalizing problems to age 8 family conflict, and from age 8 family conflict to age 10 child externalizing problems (Hypothesis #2). Bolded arrows represent primary paths of interest. For simplicity, bivariate correlations between variables at same time points and sociodemographic covariates are not shown

The current study sought to prospectively examine the bidirectional and transactional associations between family conflict and children’s caregiver-reported anxious and depressive symptoms from ages 6–10 in a socioeconomically disadvantaged, diverse sample of families, above and beyond children’s experience of cumulative victimization (see Fig. 1 for conceptual model). Given mixed evidence that family conflict may be more strongly associated with internalizing symptoms among girls (Cui et al., 2007; Kelly et al., 2016; White et al., 2014), we examined the invariance of these models across boys and girls. Additionally, as outlined further below, certain children were recruited for this study due to the presence of an official maltreatment report at or before age 4 years. Other children did not have a maltreatment report at the time of study recruitment but were nonetheless considered “high-risk” based on other sociodemographic information. In light of the potential for early maltreatment to increase vulnerability to later stressors (Hein & Monk, 2017), we also examined the invariance of these models depending on whether children were reported for maltreatment prior to study recruitment. We expected that (1) family conflict and child anxious and depressive symptoms would be associated with increases in one another bidirectionally across ages 6 and 8, and that in turn, family conflict at age 8 would predict increases in anxious and depressive symptoms at age 10; and (2) age 6 anxious and depressive symptoms would have significant indirect effects on age 10 anxious and depressive symptoms through age 8 family conflict, a process we conceptualize as transactional; (3) though the literature is limited, we also anticipated that the bidirectional associations between family conflict and anxious- and withdrawn-depressed symptoms would be stronger for girls than for boys; (4) finally, we expected that these associations would be stronger among families that were reported for maltreatment prior to study recruitment.

## Methods

### Sample and Procedures

The current study included participants from the Longitudinal Studies of Child Abuse and Neglect (LONGSCAN), a consortium of ongoing studies prospectively investigating the causes and consequences of child abuse and neglect across five sites in the Southwestern, Northwestern, Eastern, Southern and Midwestern regions of the U.S. (*N* = 1,354) Data for LONGSCAN were collected between 1993 and 2007; see (Runyan et al., 1998) for a detailed description. Participants were selected due to their involvement in Child Protective Services (CPS) as the result of a child maltreatment report (Northwest, Midwest, Southwest sites), through a state public health tracking system of high-risk families (South), or through involvement with pediatric and maternal health clinics serving low-income families (East). Each LONGSCAN site obtained approval from their respective institutional review boards and written, informed consent/assent was acquired from all participating caregivers and children.

Children and their caregivers were interviewed in-person in their homes biannually at ages 6, 8, and 10 years. The age 10 LONGSCAN interview consisted of an abbreviated battery of assessments, and as a result, interviews were conducted remotely by telephone at all sites except for the Southwestern site, which completed in-person interviews. Interviews included questions regarding the child’s development. Experiences of physical, sexual, and psychological abuse and were completed on laptop computers, using Audio Computer-Assisted Self-Interviews (ACASI) for privacy. Each LONGSCAN site also systematically reviewed CPS records continuously to identify reports of alleged maltreatment for each child/family and coded the maltreatment narratives by type and severity using the Modified Maltreatment Classification System (MMCS; English & Investigators 1997). Coders at each site were trained to use the MMCS by experienced coders until they reached 90% agreement with the gold standard. To further ensure reliable coding, coders at all five sites coded a subsample (*n* = 109) of the CPS narratives that represented cases from each site. Additionally, coders noted whether or not violence between adult family members was included in the report as a risk factor.

## Measures

### Sociodemographics

Caregivers reported on child sex at birth and race at their initial interview (0 = male, 1 = female; 0 = non-Hispanic White, 1 = non-White), and reported on other sociodemographic information (i.e., years of education, family income, number of household dependents) at each interview. We calculated an index of whether families’ compared annual income fell below the federal poverty guidelines during the years the data were collected, adjusting for number of dependents, at each interview.

### Maltreatment Prior to Recruitment

Information about the child’s maltreatment status at initial study recruitment was collected when children were age 4. A dichotomous indicator of whether (0 = No, 1 = Yes) children were reported to CPS for maltreatment prior to recruitment at age 4 was created.

### Family Conflict

Caregivers reported on their perception of family conflict within their household using the 12-item Conflict subscale of the Self-Report Family Inventory (SFI; Beavers et al., 1990) at the age 6 and 8 interviews. Data on family conflict was not collected at the age 10 interview. Respondents were asked to rate each statement (e.g., “Household members put each other down,” Grownups in the household compete with each other,” “We argue a lot and never solve problems”) on a 5-point scale ranging from 1 (fits our household very well) to 5 (doesn’t fit our household at all). An overall mean score was created, with greater scores indicating greater levels of family conflict (some items, included the aforementioned example items, are reverse-scored). The SFI has well-established psychometric properties, with reliability coefficients ranging from 0.84 to 0.88 (Beavers et al., 1985) and extensive evidence of convergent and concurrent validity (Beavers et al., 1985; Epstein et al., 1983; Hampson et al., 1991). In the current sample, internal consistency for age 6 and 8 Conflict scale was adequate (αs =. 77 and 0.72).

### Child Anxious and Depressive Symptoms

Caregivers reported on their children’s dimensional anxious and depressive symptoms using the Child Behavior Checklist (CBCL/4–18; Achenbach 1991a; Achenbach & Rescorla, 2001) at the age 6, 8, and 10 interviews. The CBCL is a widely-used, 113-item measure that assesses the frequency (0 = *not true*, 1 = *sometimes true*, 2 = *often true*) of various child behaviors during the previous six months. The current study used scores from the anxious-depressed and withdrawn-depressed syndrome scales of the CBCL (13 and 8 items, respectively). While both scales capture aspects of anxiety and depression, anxious-depressed items are comprised of both behavioral and cognitive elements, many of which represent more “outward” expressions of anxiety and depression, and include a prominent anxiety component (e.g., “Fears he/she might think or do something bad,” “Cries a lot,” “Nervous, high strung, or tense”). In contrast, withdrawn-depressed items represent more inward manifestations of depression and are strictly behavioral in nature (e.g., “Refuses to talk,” “Underactive, slow moving, or lacks energy,” “Withdrawn, doesn’t get involved with others”). The CBCL has well-established psychometric properties, with extensive evidence of reliability and validity (Achenbach et al., 2003). The syndrome scales in particular represent a dimensional, transdiagnostic assessment of anxious and depressive symptoms that align with recent efforts to explore basic dimensions of functioning and to decrease reliance on disorder-based categories (e.g., NIH’s Research Domain Criteria initiative; Insel et al., 2010). These syndrome scales were chosen over the DSM-oriented anxiety and depression scales because the latter were created post-hoc from existing CBCL items without factor-analytic methods, consist of fewer items, and do not offer a particularly compelling advantage regarding correspondence with clinical diagnoses compared to the syndrome scales (Ebesutani et al., 2010). In the current sample, internal consistency for both syndrome scales ranged from acceptable to excellent (αs = 0.70-0.91) at all three time points, with the exception of the withdrawn-depressed scale at the age 6 interview (α = 0.69), which was slightly below what is typically considered acceptable and may have been a result of the relatively low number of items on that scale (*n* = 8).

### Cumulative Victimization

Children’s cumulative victimization experiences were assessed using multi-informant, multi-modal assessments that included caregiver and CPS reports. Consistent with previous studies (e.g., Finkelhor et al., 2007; Margolin et al., 2010), four dichotomous indicators were created to identify whether or not youth had experienced (1) physical abuse, (2) sexual abuse, (3) witnessed family violence, and (4) witnessed non-family violence prior to age 6 and between ages 6 and 8. The decision to use reported allegations of maltreatment, rather than substantiated cases, was based on previous findings that children with alleged maltreatment reports are at a similarly increased risk for maltreatment recidivism and mental health consequences (Drake et al., 2003; Hussey et al., 2005; Kohl et al., 2009). The four indicators were summed to create indices (ranging from 0 to 4) of cumulative victimization between each interview. Previous researchers have provided evidence of the validity of similarly constructed cumulative victimization scores (Finkelhor et al., 2007; Margolin et al., 2010). Details about the methods and measures involved in creating the four dichotomous indicators can be found in the Supplemental Materials, as well as in previous studies (Cromer & Villodas, 2017; Morelli et al., 2020).

### Caregiver Depressive Symptoms

Caregivers’ depressive symptoms were self-reported via the Center for Epidemiologic Studies Depression Scale (CES-D) at the Age 4 and 6 interviews and via the Depression subscale of the Brief Symptom Inventory (BSI) at the age 8 interview. The CES-D and BSI are both widely used, reliable, and valid self-report measures of adult depressive symptoms, including among low-income and racially/ethnically-diverse populations (Prelow et al., 2005; Thomas et al., 2001). In the present sample, internal consistency for the CES-D was high across the Age 4 and Age 6 interviews (αs = 0.90) and moderately high for the BSI at the Age 8 interview (α = 0.83).

## Analytic Plan

Sample descriptive statistics, bivariate correlations (see Supplementary Table S1 and S2), and missing data analyses were computed using IBM SPSS version 25 (IBM Corp., 2017). Bivariate correlations were computed among all variables to identify covariates and confirmed hypothesized associations to be included in the path models. Path analyses were performed using structural equation modeling framework in M*plus* version 8 (Muthén & Muthén, 2017).

**Structural Equation Models.** M*plus* provides several indicators of overall model fit, including (1) the chi-square value (χ^2^); (2) the Comparative Fit Index (CFI; Bentler 1990); (3) the Tucker-Lewis Index (TLI; Tucker & Lewis 1973); and (4) Root Mean Square Error of Approximation (RMSEA; Steiger 1990). Based on recommendations (Hu & Bentler, 1999), a non-significant chi-square value indicated good fit, values greater than 0.95 and 0.90 indicated good and acceptable model fit, respectively, for both the CFI and TLI, and values less than 0.05 indicated acceptable fit for the RMSEA. The fit of individual paths was determined based on their statistical significance. M*plus* uses Full Information Maximum Likelihood estimation, which is a widely accepted method for handling data that can be reasonably considered Missing at Random (MAR; Enders 2010).

**Fig. 2:**
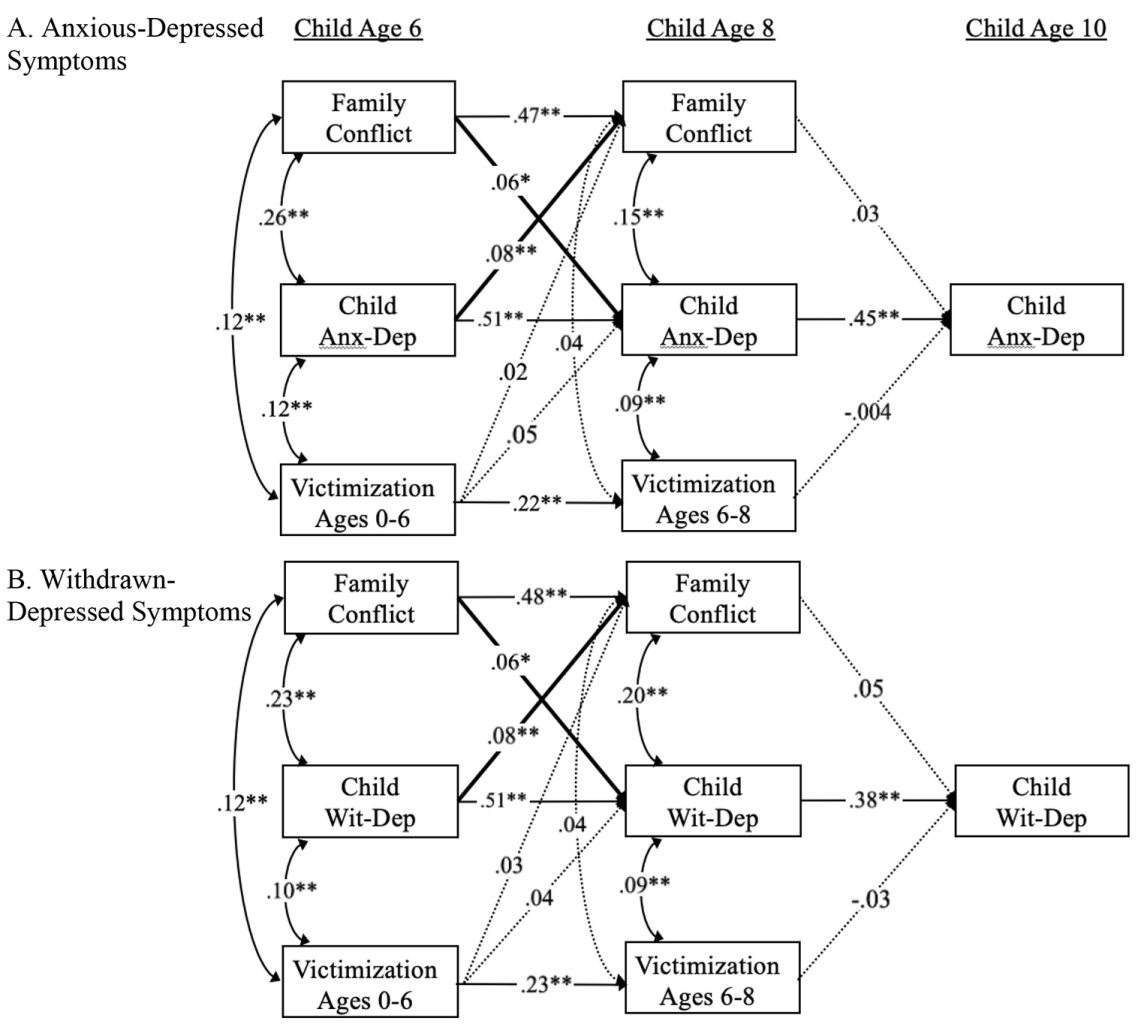
Structural path models with standardized path coefficients depicting cross-lagged bidirectional and transactional associations between family conflict and anxious-depressed symptoms (A; top) and withdrawn-depressed symptoms (B; bottom). Anx-Dep = anxious-depressed symptoms; Wit-Dep. = withdrawn-depressive symptoms; solid arrows represent significant paths; dotted arrows represent nonsignificant paths; bolded arrows represent significant paths in support of primary hypotheses. For simplicity, paths involving sociodemographic covariates are not shown. *p < .05; **p < .01

To address our primary objective of examining the nature and directionality of associations between family conflict and children’s anxious and depressive symptoms, we tested two separate path analytic models, one with withdrawn-depressed and the other with anxious-depressed symptoms (Fig. 2). In each model, all autoregressive and cross-lagged paths between subsequent measurement occasions were freely estimated. Correlations were specified between variables within measurement occasions. Time-invariant covariates (i.e., child race/ethnicity, gender, caregiver years of education, recruitment status) were included as predictors of all endogenous variables in the model. Poverty and cumulative victimization were included as time-varying covariates predicting levels of family conflict and anxious- and depressive symptoms at subsequent timepoints. Of note, anxious- and withdrawn-depressed symptoms were assessed using the CBCL, which probes behavior problems within the past six months. Given potential overlap with cumulative victimization (e.g., cumulative victimization between ages 0–6 could occur after caregivers’ report of anxious- or withdrawn-depressive symptoms at the age 6 interview), cumulative victimization between ages 0–6 and 6–8 were included as time-varying predictors of age 8 and age 10 anxious-/withdrawn-depressed symptoms and family conflict, respectively. Associations between potentially overlapping victimization and anxious-/withdrawn-depressed symptoms (e.g., victimization from 0 to 6 and anxious-/withdrawn-depressed symptoms at age 6) were specified as concurrent associations. Finally, sensitivity analyses tested caregiver depressive symptoms at prior timepoints as a predictor of family conflict and child symptoms to examine whether caregivers’ own symptomatology may have impacted model fit and hypothesized paths of interest.

To test our secondary objective of examining transactional processes from age 6 anxious- and withdrawn-depressed symptoms to age 10 anxious- and withdrawn-depressed symptoms through age 8 family conflict, we first examined the joint significance of the path coefficients between the predictor (i.e., each age 6 symptom domain) and mediator variable (i.e., age 8 family conflict; path *a*) and the mediator and outcome variables (each age 10 symptom domain; path b; MacKinnon et al., 2002). If both paths were significant, we would then calculate indirect effects based on the product of the unstandardized path coefficients. The significance of indirect effects was determined using bias-corrected 95% confidence intervals (CIs) based on 1000 bootstrapped samples (Preacher & Hayes, 2008). Tests of model invariance were conducted to determine whether or not the proposed models were moderated by gender or presence of an official maltreatment allegation prior to study recruitment (i.e., between child ages 0–4). We compared the fit of a model in which all parameters were freely estimated for both groups separately (i.e., unconstrained model) to the fit of nested models in which all or a subset of the parameters were constrained to equivalence across groups. If a fully constrained model fit the data significantly worse than the unconstrained model, based on the χ^2^ difference (Δχ^2^) test, model modification indices (i.e., Lagrange Multiplier test) were examined to determine which parameters contributed most strongly to the non-invariance (i.e., which parameters differed between groups). These parameter constraints were then iteratively removed until the partially constrained model fit did not significantly differ from the unconstrained model fit (i.e., Δχ^2^*p* > .05).

## Results

### Missing Data

Seventy-three (5%) of the total 1,354 caregiver-child dyads who participated in LONGSCAN were not interviewed at any of the three time points included in the study. This resulted in a final sample size of 1,281 for the current study. Of these, 898 (70%) were interviewed at all three time points (i.e., child age 6, 8, and 10). The number of participants who completed interviews at each time point were 1,225 (90.5%) at age 6, 1,074 (79%) at age 8, and 1,018 (75%) at age 10. Proportions of participants with missing data ranged from 4.9% for children’s anxious- and withdrawn-depressive symptoms at age 6 to 22% for children’s anxious- and withdrawn-depressive symptoms at age 10. Little’s (1988) test of missing data patterns was not significant, χ^2^(92) = 101.95, *p* > .05, indicating that the data can be treated as though they were Missing Completely At Random (MCAR). Additionally, participants who missed one or more interviews did not differ from those with complete data on gender, cumulative victimization, poverty, or anxious- or withdrawn-depressed symptoms (*p*s > 0.05) at initial recruitment (i.e., at four years of age). However, those who missed at least one interview were disproportionately non-White, χ^2^(1) = 9.67, *p* = .002, and were less likely to have been recruited due to a CPS maltreatment allegation prior to age 4, χ^2^(1) = 52.34, *p* < .001. Nevertheless, these data can still be considered MAR (Enders, 2010) and these factors were included as covariates in subsequent models.

## Sample Characteristics and Bivariate Correlations

Mean family conflict, rated on a Likert-type scale from 1 to 5, was 1.69 and 1.71 and at the child age 6 and 8 interviews. This figure is slightly lower than expected given the high-risk nature of the LONGSCAN sample. A sizable percentage of children were exposed to one or more types of violent victimization between age 0 and 6 years (20.6%) and between 6 and 8 (11.5%). Children’s anxious- and withdrawn-depressive symptoms were slightly elevated relative to normative populations, with mean *T*-scores between 53.98 and 55.67. All other sociodemographic information and descriptive statistics can be found in Table S1. All covariates were associated with at least one endogenous variable in each model; all of their predictive paths were included in the final models. Bivariate correlations for study variables can be found in Supplementary Table S2; the effects of covariates on primary variables of interest can be found in Table S3.

## Bidirectional Models of Family Conflict and Anxious-Depressed Symptoms

The bidirectional model predicting children’s anxious-depressed symptoms fit the data well, χ^2^(15) = 17.39, *p* = .30, CFI = 0.99, TLI = 0.99, RMSEA = 0.01. Consistent with prior research, autoregressive coefficients across contiguous time points were moderate-to-strong in magnitude for family conflict, β = 0.47, *p* < .001, and anxious-depressed symptoms, βs = 0.45-0.51, *p*s < 0.001. The autoregressive coefficient for cumulative victimization between ages 0–6 and 6–8 was moderate in magnitude, β = 0.22, *p* < .001. After controlling for prior cumulative victimization, paths between family conflict and anxious-depressed symptoms revealed a cross-lagged, bidirectional pattern, such that more age 6 family conflict was associated with increases in age 8 anxious-depressed symptoms, β = 0.06, *p* = .04, while more age 6 anxious-depressed symptoms were associated with increases in age 8 family conflict, β = 0.08, *p* = .03 (Fig. 2 A). Family conflict at age 8 was not significantly associated with anxious-depressed symptoms at age 10, β = 0.03, *p* = .23. As such, indirect effects were not tested in this model. Cumulative victimization was generally associated with family conflict and anxious-depressed symptoms at concurrent time points, but not at distal time points. Sensitivity analyses revealed that caregiver depressive symptoms at age 8 were significantly associated with increases in child anxious-depressive symptoms at age 10 (β = 0.09, *p* = .02). Caregiver depressive symptoms were not significantly associated with family conflict or child symptoms at any other timepoints. Including caregiver depressive symptoms in the models did not change the significance of any of the hypothesized paths described above.

## Invariance of Bidirectional Models Predicting Children’s Anxious-Depressed Behaviors

### Gender

Tests of model invariance revealed that the fully constrained model did not fit the data significantly worse than the unconstrained model, Δχ^2^(59) = 71.16, *p* = .13, suggesting that the bidirectional associations identified in the full sample functioned similarly for boys and girls. (Table 1). No further invariance tests were conducted.

**Table 1:** Fit Indices for Tests of Model Invariance

Model	χ^2^	df	CFI	TLI	RMSEA	Δχ^2^	Δdf
**Gender**							
Anxious-Dep Symptoms							
Unconstrained	20.34	12	0.99	0.96	0.04	-	-
Fully Invariant	87.07	71	0.99	0.99	0.02	71.16	59
Withdrawn-Dep Symptoms							
Unconstrained	14.87	12	0.99	0.98	0.02	-	-
Fully Invariant	87.72	71	0.99	0.98	0.02	73.56	59
**Pre-recruitment Maltreatment**							
Anxious-Dep Symptoms							
Unconstrained	16.83	12	0.99	0.97	0.03	-	-
Fully Invariant	97.60*	71	0.99	0.97	0.03	82.10*	59
Partially Invariant 1^a^	84.93	70	0.99	0.99	0.02	70.16	58
Withdrawn-Dep Symptoms							
Unconstrained	4.64	12	1.00	1.05	0.00	-	-
Fully Invariant	110.52**	71	0.98	0.96	0.03	100.07***	59
Partially Invariant 1^b^	92.60*	70	0.99	0.98	0.02	83.04*	58
Partially Invariant 2^c^	83.56	69	0.99	0.98	0.02	74.63	57

*Note*. df = degrees of freedom; CFI = comparative fit index; TLI = Tucker–Lewis index; RMSEA = root-mean-square error of approximation; a = autoregressive path for anxious-depressive symptoms at ages 8 and 10; b = autoregressive path for withdrawn-depressive symptoms at ages 8 and 10; c = path between caregiver years of education and age 6 withdrawn-depressive symptoms

### Pre-Recruitment Maltreatment Status

Tests of model invariance revealed that the fully constrained model fit the data significantly worse than the unconstrained model across participants who did vs. did not have child maltreatment reports prior to study recruitment, Δχ^2^(59) = 82.10, *p* = .03 (Table 1). Guided by model modification indices, one parameter constraint was removed before a partially constrained model fit the data as well as the unconstrained model. The constraint that was removed was for the autoregressive path for anxious-depressive symptoms between ages 8 and 10. Though it was significant in both groups, it was stronger among those with a pre-recruitment maltreatment report, β = 0.52, *p* < .001 compared to those without a report, β = 0.34, *p* < .001.

## Bidirectional Models of Family Conflict and Withdrawn-Depressed Symptoms

The bidirectional model predicting children’s withdrawn-depressed symptoms fit the data well, χ^2^(15) = 12.12, *p* = .67, CFI = 1.00, TLI = 1.00, RMSEA = 0.00. Autoregressive coefficients across contiguous time points were moderate-to-strong in magnitude for family conflict, β = 0.46, *p* < .001, and withdrawn-depressed symptoms, βs = 0.37-0.56, *p*s < 0.001. The autoregressive coefficient for cumulative victimization between ages 0–6 and 6–8 was moderate in magnitude, β = 0.18, *p* < .001. Similar to the model predicting anxious-depressed symptoms, after controlling for prior cumulative victimization, paths between family conflict and withdrawn-depressed symptoms revealed a cross-lagged, bidirectional pattern, such that more age 6 family conflict was associated with increases in age 8 withdrawn-depressed symptoms, β = 0.06, *p* = .04, while more age 6 withdrawn-depressed symptoms were associated with increases in age 8 family conflict, β = 0.08, *p* = .01 (Fig. 2B). Family conflict at age 8 was not significantly associated with withdrawn-depressed symptoms at age 10, β = 0.05, *p* = .12. As such, indirect effects were not tested. Cumulative victimization was generally associated with family conflict and withdrawn-depressed symptoms at concurrent time points, but not at distal time points.

## Invariance of Bidirectional Models Predicting Children’s Withdrawn-Depressed Behaviors

### Gender

Tests of model invariance revealed that the fully constrained model did not fit the data significantly worse than the unconstrained model, Δχ^2^(59) = 73.56, *p* = .10, suggesting once again that the bidirectional associations identified in the full sample functioned similarly in boys and girls (Table 1). No further invariance tests were conducted.

### Pre-Recruitment Maltreatment Status

Tests of model invariance revealed that the fully constrained model fit the data significantly worse than the unconstrained model across participants who did vs. did not have child maltreatment reports prior to study recruitment, Δχ^2^(59) = 82.10, *p* = .03 (Table 1). Guided by model modification indices, two parameter constraints were iteratively removed before a partially constrained model fit the data as well as the unconstrained model. These removed constraints were for (1) the autoregressive path for withdrawn-depressive symptoms across ages 8 and 10 and (2) the path between caregiver years of education and age 6 withdrawn-depressive symptoms. Though the autoregressive path for withdrawn-depressive symptoms across ages 8 and 10 was significant in both groups, it was stronger among those with a pre-recruitment maltreatment report, β = 0.40, *p* < .001, compared to those without a report, β = 0.20, *p* < .001. Also, the path between caregiver years of education and age 6 withdrawn-depressive symptoms was negative and significant for those without a pre-recruitment maltreatment report, β = − 0.17, *p* < .001, and nonsignificant for those with a report, β = − 0.01, *p* = .73.

## Discussion

Everyday patterns of family conflict represent a chronic environmental stressor for children. Family systems theorists have long emphasized the importance of modeling bidirectional relationships involving the broader family (Davies & Sturge-Apple, 2014; Persram et al., 2019). The present study investigated the bidirectional associations between family conflict and two key dimensional subdomains of internalizing psychopathology across middle childhood (i.e., ages 6, 8, and 10), controlling for cumulative victimization, in a sample of families with or at high risk for violence exposure and perpetration. Findings revealed a similar pattern of cross-lagged, bidirectional associations between family conflict and anxious- and withdrawn-depressed symptoms across child ages 6 to 8 that were mostly invariant across gender and early maltreatment history. Family conflict was not associated with child internalizing psychopathology at age 10. This investigation is the first known attempt to model bidirectional associations between family conflict and child psychopathology above and beyond the effects of more severe interpersonal violence.

## Bidirectional Associations and Transactional Processes

The cross-lagged associations identified here suggest a problematic process in which children’s anxious- and withdrawn-depressed symptoms and families’ negative patterns of interaction exacerbate one another over time, particularly in early and middle childhood. The prospective link from family conflict to child anxious- and withdrawn-depressed symptoms from ages 6 to 8 aligns with decades of research indicating that family dysfunction increases risk for the development of children’s mood and anxiety problems (Anyan & Hjemdal, 2018; Au et al., 2009; Jozefiak & Wallander, 2016; Kelly et al., 2016; Negy & Snyder, 2006; Rabinowitz, Osigwe, et al., 2016; Rapp et al., 2017). Also consistent with prior research was the relatively high stability of child psychopathology and family conflict over time (Haberstick et al., 2005; Stadelmann et al., 2010). Although the present investigation centered on reciprocal family-child effects, trait levels of child internalizing tendencies and family characteristics should not be ignored.

The prospective link from children’s anxious- and withdrawn-depressive symptoms to family conflict, on the other hand, represents a more a novel finding. Children’s internalizing symptoms often reflect disengaged and withdrawn coping strategies, behaviors that have been associated with increased stress from family members (Roubinov & Luecken, 2013; Rubin & Mills, 1992). Relatedly, although behavioral expressions of anxiety and depression may initially elicit support, repeated negative self-statements, reassurance seeking, school refusal, and overall impaired social functioning may lead to rejection or frustration from family members, increasing conflict within the household (Joiner & Coyne, 1999). Ultimately, more research is needed to replicate these findings and to explore mediating pathways, including how internalizing behaviors contribute on a day-to-day level to the long-term bidirectional patterns identified here. Nevertheless, our findings add to the growing body of literature indicating that children’s internalizing symptoms could conceivably exert a negative influence within the family system. We emphasize that directional effects would likely be attributable to maladaptive responses from family members (e.g., difficulty regulating frustration and stress) than to children themselves.

The prospective pathways from family conflict to anxious- and withdrawn-depressed symptoms were significant across child ages 6 and 8, but not across ages 8 and 10. Accordingly, a transactional process from age 6 symptoms to age 10 symptoms through age 8 family conflict was not supported. Similar transactional models have been identified elsewhere, including a process linking age 13 depressive symptoms with age 15 depressive symptoms through age 14 family conflict (Kelly et al., 2016). It is unclear why the family conflict-to-internalizing symptom paths tested in the present study were significant in between ages 6 and 8, but not ages 8 and 10. It is possible that arguing, fighting, and criticism between family members is perceived as more stressful or dangerous by younger children, particularly in family contexts where risk for violence is escalated. Young children may not be able to distinguish lower-risk negative interactions from more dangerous ones, resulting in further chronic reactivation of stress response systems and increasing risk for the development of mood and anxiety problems (Simpson et al., 2012). Additionally, older children may be able to better leverage certain protective factors not assessed in the present study (e.g., peer support), potentially benefiting from a buffering effect not yet available to younger children (Wren, 2020). Alternatively, this null finding may reflect a statistical artifact.

Contrary to expectations, invariance testing revealed no significant differences in model fit across gender. A handful of studies investigating similar bidirectional models in adolescence also did not identify gender differences (Kelly et al., 2016; Saxbe et al., 2014; Timmons & Margolin, 2015). Yet, other literature suggests that girls may be particularly vulnerable to the development of internalizing problems following family-related conflict (Crawford et al., 2001; Essex et al., 2003). Future work should explore how family conflict might impact other domains of psychopathology in gender-specific ways (e.g., boys’ externalizing symptoms; Feiring et al., 1999; Gray & Rarick, 2018). Invariance testing across pre-recruitment maltreatment history revealed minimal differences which involved paths that were unrelated to the study’s primary research questions. In sum, the bidirectional models identified in the current study functioned similarly among boys and girls and participants with and without early maltreatment reports. The models also did not change meaningfully after including caregiver depressive symptoms as predictors of family conflict and child symptoms prior to each time point, indicating that the bidirectional associations identified here were not an artifact of caregivers’ own symptomatology or depressive style of reporting.

## Strengths and Limitations

The present study maintains several key strengths. We extend previous work on bidirectional family-child effects in a large, racially/ethnically diverse, high-risk sample, while leveraging prospective, longitudinal data spanning several years. We also shed novel insight on the potentially crucial role of family conflict, independent of the most severe forms of interpersonal victimization, on children’s socioemotional development. By doing so, we provide support for family systems frameworks (e.g., holism), which posit that the family unit is not reducible to an additive sum of functioning within each individual or dyad (Cox et al., 2001).

Nevertheless, limitations of this study should be noted. First, while some indicators of cumulative victimization were assessed using gold-standard, multi-informant assessments (i.e., CPS records and caregiver reports of physical abuse), assessment of whether children witnessed non-family violence was limited to caregivers, who may be less knowledgeable about violence their children are exposed to outside of the home (Goodman et al., 2010). A second limitation of this study is that several variables of interest – children’s anxious- and withdrawn-depressive symptoms and family conflict – were limited to caregivers’ reports; LONGSCAN did not administer youth-report measures of psychopathology or family dynamics until adolescence. Caregivers in the current study, many of whom were involved with CPS, may have underreported levels of family conflict to protect their families from unwanted scrutiny. Additionally, internalizing problems are sometimes considered to be more valid when self-reported depending on the child’s age (De Los Reyes et al., 2015). More critically, caregivers’ perception of family conflict and their children’s anxious- and depressive symptoms may be associated as a result of confounding reasons, such as reporting style, cognitive biases, or genetic predispositions that underly both family conflict and anxious and depressive symptoms, each of which could magnify the associations identified in the current study (Haworth et al., 2017). For these reasons, future work should consider both caregiver and child reports of family conflict and internalizing symptoms and account for potential genetic influence to better contextualize findings. Second, family conflict in LONGSCAN was not assessed at the age 10 interview. As a result, cross-lagged associations from age 8 psychopathology to age 10 family conflict were not examined, nor were transactional processes from age 6 family conflict to age 10 family conflict through age 8 psychopathology. Third, the data on psychopathology were collected between March 1993 and July 2007. The cohort of children spanned a wide range, but it is possible that these data are limited by cohort changes in anxiety subsequent to this period (Collishaw, 2015). Finally, children were recruited based on their high risk for maltreatment exposure, and therefore constituted a particularly high-risk group whose results may not generalize to lower-risk, higher-SES families more representative of the broader U.S. population.

## Implications

Findings from the present study provide a number of implications for clinical practice and future research. First, we provide evidence that interventions aiming to improve child emotional problems by targeting severely dysfunctional family processes (Chen & Chan, 2016; Mikton & Butchart, 2009) should also address non-violent, lower-level patterns of negative family interactions. This may be particularly crucial for children who, as a product of prior victimization, may be especially susceptible to the negative effects of nonviolent family conflict (Toth & Cicchetti, 2013). A second clinical implication relates to our focus on child anxious- and withdrawn-depressive symptoms rather than externalizing symptoms. Prominent theoretical models of bidirectional family-child effects typically describe the development of children’s aggressive or disruptive behaviors (e.g., Coercive Family Process; Patterson 1982). Similarly, parent-mediated interventions (e.g., Parent Management Training/Behavioral Parent Training) typically seek to minimize escalations involving child externalizing behaviors and family members’ maladaptive responses by teaching selective attention and ignoring. Our findings suggest that children’s internalizing behaviors may also increase stress within the family system, which may require a different set of parent-mediated strategies to break the problematic bidirectional patterns identified here. Regarding future directions for research, our findings lay the groundwork for studies to examine the day-to-day or minute-to-minute family-child interactions that might contribute to the long-term reciprocal associations found across the Age 6 and 8 time points (e.g., Timmons & Margolin 2015). Related, future studies could examine whether the reciprocal impact of family conflict and internalizing problems is specific to earlier developmental periods, as was the case here. Finally, given the strong temporal stability of family conflict and child psychopathology that we observed, future work with similar panel data might benefit from modeling the within-person and between-person variance separately. Though outside the scope of the present study, random intercept cross-lagged panel modeling could be useful for understanding how temporal fluctuations in the family environment and in child psychopathology impact each other after accounting for stable, trait levels of these constructs (Hamaker et al., 2015).

## Conclusion

We identified key patterns of bidirectionality across ages 6 and 8 for both anxious- and withdrawn-depressive symptoms. These patterns were largely similar across boys and girls and for children with and without early maltreatment reports. Transactional processes from age 6 to age 10 anxious- and withdrawn-depressive symptoms through age 8 family conflict were not supported. These findings provide novel insight into the reciprocal nature of child development and everyday family processes in early and middle childhood over and above violent victimization and suggest the possibility that children’s internalizing symptoms, in certain contexts, may influence the family system, an area that requires further investigation and understanding. This work supports early theoretical models (e.g., Bell 1968; Belsky, 1984; Crouter & Booth, 2003) and expands upon more recent empirical work (e.g., Burt et al., 2005; Choe & Zimmerman, 2014; Kelly et al., 2016) suggesting that children actively influence their most proximal social environments, particularly in early and middle childhood.

## Electronic Supplementary Material

Below is the link to the electronic supplementary material.

Supplementary Material 1
