# Supplementary material for: Everyday Conflict in Families at Risk for Violence Exposure: Examining Unique, Bidirectional Associations with Children’s Anxious- and Withdrawn-Depressed Symptoms
Source: Res Child Adolesc Psychopathol. 2022 Nov 4;51(3):317–30. doi: 10.1007/s10802-022-00966-6 (PMC9908649; doi:10.1007/s10802-022-00966-6)
Supplement: Supplementary file 1 — Supplementary Material 1 [file 10802_2022_966_MOESM1_ESM.docx]

**Supplemental Materials**

**Description of Methods and Measures Involved in Creation of Cumulative Victimization Indicators (Physical Abuse, Sexual Abuse, Witnessed Family Violence, Witnessed Non-Family Violence):**

**Physical abuse.** Indicators of allegations of physical abuse were included based on systematic reviews of CPS reports, as described above, before the age 6 interview and between age 6 and age 8 interviews. Caregivers also reported their own assaultive behavior toward their children in the past year at the age 8 interview on the severe assault scale of the Conflict Tactics Scale-Parent-Child version (CTSPC; Straus, Hamby, Finkelhor, Moore, & Runyan, 1998). The severe assault scale of the CTSPC includes six items, including kicking, biting, burning, and scalding the child. Although this scale has demonstrated good validity, it tends to show weak evidence of internal consistency because of the relatively low frequency of severe behaviors (Straus et al., 1998). CPS and caregiver-reported physical abuse measures were combined into single dichotomous indicators (i.e., 0 = not alleged, 1= alleged) signifying whether or not any physical abuse allegedly occurred between children’s ages 0-6 and 6-8 years.

**Sexual Abuse.** Indicators of allegations of sexual abuse were included based on systematic reviews of CPS reports, as described above. A single dichotomous indicator (i.e., 0 = not alleged, 1= alleged) signified whether or not any sexual abuse allegedly occurred between children’s ages 0-6 and 6-8 years.

**Witnessed Family Violence.** Caregivers completed the Child’s Life Events Scale (LES), adapted by LONGSCAN from Coddington’s (1972) Life Event Records to include items more representative of youth from low-income and child welfare samples, at the age 6 and 8 interviews. Caregivers reported the number of times youth had witnessed family members involved in any of five acts of violence in the past year, including someone being: threatened with a weapon; hit, kicked, or slapped; shot or stabbed; raped or sexually assaulted; killed or murdered. Additionally, information from CPS reports that included domestic violence as a risk factor between ages 0-6 or 6-8 were included as indicators of family violence during those time periods, as has been done in previous LONGSCAN analyses (English, Marshall, & Stewart, 2003). CPS reports and caregiver reports were combined into single dichotomous indicators (i.e., 0 = not alleged, 1 = alleged) of witnessed family violence between ages 0-6 and 6-8.

**Witnessed Non-Family Violence.** Witnessing non-family violence was assessed solely based on caregiver report via the LES (described above) at the age 6 and 8 interviews, which indicated the number of times youth witnessed non-family members involved in any of five acts of violence in the past year, including someone being: threatened with a weapon; hit, kicked, or slapped; shot or stabbed; raped or sexually assaulted; killed or murdered. Single dichotomous indicators (i.e., 0 = not alleged, 1 = alleged) were formed based on the LES signifying whether or not children witnessed non-family violence in the past year at age 6 or at age 8.

**Table S1**

*Sociodemographic Information and Descriptive Statistics*

|  | Full Sample  *N* = 1,281 | Girls  *n* = 658  (51.4%) | Boys  *n* = 623  (48.6%) | No Early Maltreatment  *n* = 521 (40.7%) | Early  Maltreatment  *n* = 760 (59.3%) |
| --- | --- | --- | --- | --- | --- |
| Below federal poverty level (age 0-6); N (%) | 920 (71.8%) | 467 (71.0%) | 453 (72.7%) | 408 (78.3%) | 512 (67.4%) |
| Below federal poverty level (age 8); N (%) | 634 (49.5%) | 324 (49.2%) | 310 (49.8%) | 297 (57.0%) | 337 (44.3%) |
| Caregiver years of education (youth age 6); *M* (SD) | 11.76 (2.19) | 11.78 (2.13) | 11.74 (2.26) | 11.38 (1.83) | 12.02 (2.37) |
| Race: White; N (%) | 325 (25.4%) | 164 (24.9%) | 161 (25.8%) | 81 (15.5%) | 244 (32.1%) |
| Race: Non-White; N (%) | 956 (74.6%) | 494 (75.1%) | 461 (74.0%) | 440 (84.5%) | 516 (67.9%) |
| # of People Living in Household (age 6); *M* (SD) | 4.88 (1.87) | 4.98 (1.95) | 4.77 (1.79) | 4.64 (1.68) | 5.04 (1.98) |
| # of People Living in Household (age 8); *M* (SD) | 4.92 (1.91) | 4.94 (2.05) | 4.91 (1.75) | 4.66 (1.66) | 5.11 (2.04) |
| Family Conflict Mean Score (age 6); *M* (SD) | 1.71 (0.64) | 1.69 (0.64) | 1.73 (0.64) | 1.73 (0.68) | 1.70 (0.62) |
| Family Conflict Mean Score (age 8); *M* (SD) | 1.69 (0.61) | 1.64 (0.58) | 1.74 (0.65) | 1.66 (0.61) | 1.71 (0.62) |
| Cumulative Victimization (ages 0-6); *M* (SD) | 0.72 (0.94) | 0.72 (0.95) | 0.72 (0.93) | 0.32 (0.66) | 1.00 (1.00) |
| Cumulative Victimization (age 6-8); *M* (SD) | 0.47 (0.75) | 0.44 (0.74) | 0.51 (0.76) | 0.40 (0.66) | 0.52 (0.81) |
| CBCL Anxious-Dep. T-Score (age 6); *M* (SD) | 53.98 (6.29) | 53.93 (6.07) | 54.04 (6.51) | 53.23 (5.39) | 54.49 (6.78) |
| CBCL Anxious-Dep. T-Score (age 8); *M* (SD) | 54.67 (6.95) | 54.26 (6.35) | 55.12 (7.53) | 53.58 (6.12) | 55.40 (7.37) |
| CBCL Anxious-Dep. T-Score (age 10); *M* (SD) | 54.40 (7.11) | 53.73 (6.54) | 55.14 (7.63) | 52.78 (5.57) | 55.25 (7.66) |
| CBCL Withdrawn-Dep. T-Score (age 6); *M* (SD) | 55.28 (6.65) | 55.35 (6.83) | 55.20 (6.46) | 54.63 (5.86) | 55.71 (7.11) |
| CBCL Withdrawn-Dep. T-Score (age 8); *M* (SD) | 55.67 (7.21) | 55.44 (7.13) | 55.91 (7.30) | 55.17 (6.70) | 56.55 (7.48) |
| CBCL Withdrawn-Dep. T-Score (age 10); *M* (SD) | 55.08 (7.04) | 54.60 (6.79) | 55.63 (7.30) | 53.49 (5.49) | 57.71 (7.38) |

*Note. M* = mean; SD = Standard Deviation; CBCL = Child Behavior Checklist; Dep = Depressed; “Early Maltreatment” and “No Early Maltreatment” indicates presence and absence, respectively, of an official maltreatment allegation prior to recruitment at child age 4 years.

**Table S2**

*Bivariate Correlations among Study Variables*

|  | 1 | 2 | 3 | 4 | 5 | 6 | 7 | 8 | 9 | 10 | 11 | 12 | 13 | 14 | 15 |
| --- | --- | --- | --- | --- | --- | --- | --- | --- | --- | --- | --- | --- | --- | --- | --- |
| 1. Gender | — |  |  |  |  |  |  |  |  |  |  |  |  |  |  |
| 2. Race^a^ | -.011 | — |  |  |  |  |  |  |  |  |  |  |  |  |  |
| 3. Education years^a^ | .010 | .046 | — |  |  |  |  |  |  |  |  |  |  |  |  |
| 4. Maltreatment status^b^ | .002 | .187** | .142** | — |  |  |  |  |  |  |  |  |  |  |  |
| Age 6 |  |  |  |  |  |  |  |  |  |  |  |  |  |  |  |
| 5. Poverty | -.034 | -.204** | -.315** | -.122** | — |  |  |  |  |  |  |  |  |  |  |
| 6. Polyvictimization | .004 | .197** | .044 | .355** | .018 | — |  |  |  |  |  |  |  |  |  |
| 7. Conflict | -.030 | .051 | -.140** | -.023 | .091** | .108** | — |  |  |  |  |  |  |  |  |
| 8. Anxious-depressed | .031 | .096** | -.003 | .103** | -.045 | .172** | .264** | — |  |  |  |  |  |  |  |
| 9. Withdrawn-depressed | .054 | .060* | -.047 | .073* | .005 | .131** | .233** | .639** | — |  |  |  |  |  |  |
| Age 8 |  |  |  |  |  |  |  |  |  |  |  |  |  |  |  |
| 10. Poverty | -.014 | -.232** | -.261** | -.151** | .433** | -.062* | .074* | -.022 | .017 | — |  |  |  |  |  |
| 11. Polyvictimization | -.050 | -.004 | -.054 | .081** | .119** | .239** | .125** | .112** | .075** | .104** | — |  |  |  |  |
| 12. Conflict | -.080** | .076* | -.086** | .040 | .079** | .107** | .499** | .209** | .194** | .053 | .125** | — |  |  |  |
| 13. Anxious-depressed | -.012 | .145** | .016 | .124** | -.036 | .173** | .202** | .542** | .402** | -.018 | .159** | .257** | — |  |  |
| 14. Withdrawn-depressed | .011 | .054 | .011 | .056 | .011 | .119** | .180** | .392** | .515** | .023 | .140** | .269** | .659** | — |  |
| Age 10 |  |  |  |  |  |  |  |  |  |  |  |  |  |  |  |
| 15. Anxious-depressed | -.057 | .137** | .083* | .174** | -.053 | .202** | .138** | .469** | .342** | -.023 | .094** | .203** | .589** | .410** | — |
| 16. Withdrawn-depressed | -.039 | .054 | .027 | .164** | -.017 | .123** | .126** | .335** | .429** | -.029 | .052 | .196** | .392** | .501** | .703** |

*Note.*^a^0 = non-White, 1 = White*,* ^b^caregiver education years; ^c^0 = recruited based on CPS maltreatment allegation, 1 = recruited based on high-risk for maltreatment.

**p* < .05. ***p* < .01.

**Table S3**

**Covariate Effects on Variables of Interest for Anxious-Depressive and Withdrawn-Depressive Models**

|  | **Model Predicting Anxious-Depressive Symptoms** | | **Model Predicting Withdrawn-Depressive Symptoms** | |
| --- | --- | --- | --- | --- |
|  | β | *p-*value | β | *p-*value |
| Gender 🡪 Internalizing Behavior Age 6 | 0.02 | 0.41 | 0.05 | 0.08 |
| Education Years^b^ 🡪 Internalizing Behavior Age 6 | -0.02 | 0.46 | -0.06 | **0.03** |
| Maltreatment Status^c^ 🡪 Internalizing Behavior Age 6 | 0.09 | **0.002** | 0.07 | **0.01** |
| Race^a^ 🡪 Internalizing Behavior Age 6 | 0.08 | **0.005** | 0.05 | 0.10 |
| Gender 🡪 Family Conflict Age 6 | -0.03 | 0.24 | -0.03 | 0.26 |
| Education Years^b^ 🡪 Family Conflict Age 6 | -0.14 | **<0.001** | -0.14 | **<0.001** |
| Maltreatment Status^c^ 🡪 Family Conflict Age 6 | -0.01 | 0.63 | -0.02 | 0.61 |
| Race^a^ 🡪 Family Conflict Age 6 | 0.06 | **0.04** | 0.06 | **0.04** |
| Education Years^b^ 🡪 Cumulative Victimization 0-6 | -0.01 | 0.81 | -0.01 | 0.80 |
| Maltreatment Status^c^ 🡪 Cumulative Victimization 0-6 | 0.33 | **<0.001** | 0.33 | **<0.001** |
| Race^a^ 🡪 Cumulative Victimization 0-6 | 0.14 | **<0.001** | 0.14 | **<0.001** |
| Gender 🡪 Internalizing Behavior Age 8 | -0.02 | 0.43 | -0.01 | 0.81 |
| Poverty 0-6 🡪 Internalizing Behavior Age 8 | 0.01 | 0.76 | 0.01 | 0.68 |
| Education Years^b^ 🡪 Internalizing Behavior Age 8 | 0.01 | 0.76 | 0.31 | 0.28 |
| Maltreatment Status^c^ 🡪 Internalizing Behavior Age 8 | 0.03 | 0.22 | -0.01 | 0.82 |
| Race^a^ 🡪 Internalizing Behavior Age 8 | 0.07 | **0.01** | 0.02 | 0.41 |
| Gender 🡪 Family Conflict Age 8 | -0.06 | **0.03** | -0.06 | **0.02** |
| Poverty 0-6 🡪 Family Conflict Age 8 | 0.04 | 0.13 | 0.04 | 0.18 |
| Education Years^b^ 🡪 Family Conflict Age 8 | -0.02 | 0.42 | -0.03 | 0.40 |
| Maltreatment Status^c^ 🡪 Family Conflict Age 8 | 0.03 | 0.21 | 0.04 | 0.20 |
| Race^a^ 🡪 Family Conflict Age 8 | 0.05 | 0.07 | 0.05 | 0.06 |
| Education Years^b^ 🡪 Cumulative Victimization 6-8 | -0.02 | 0.58 | -0.02 | 0.58 |
| Maltreatment Status^c^ 🡪 Cumulative Victimization 6-8 | 0.02 | 0.48 | 0.02 | 0.43 |
| Race^a^ 🡪 Cumulative Victimization 6-8 | -0.04 | 0.16 | -0.04 | 0.18 |
| Gender 🡪 Internalizing Behavior Age 10 | -0.04 | 0.13 | -0.04 | 0.19 |
| Poverty 6-8 🡪 Internalizing Behavior Age 10 | 0.01 | 0.63 | -0.03 | 0.29 |
| Education Years^b^ 🡪 Internalizing Behavior Age 10 | 0.05 | 0.06 | -0.004 | 0.90 |
| Maltreatment Status^c^ 🡪 Internalizing Behavior Age 10 | 0.09 | **0.001** | 0.12 | **<0.001** |
| Race^a^ 🡪 Internalizing Behavior Age 10 | 0.03 | 0.27 | -0.01 | 0.70 |

*Note.*^a^0 = non-White, 1 = White*,* ^b^caregiver education years; ^c^0 = recruited based on CPS maltreatment allegation, 1 = recruited based on high-risk for maltreatment.

References

Coddington, R. D. (1972). The significance of life events as etiologic factors in the diseases of children—II a study of a normal population. *Journal of Psychosomatic Research, 16*(3), 205-213. doi:10.1016/0022-3999(72)90045-1

English, D. J., Marshall, D. B., & Stewart, A. J. (2003). Effects of family violence on child behavior and health during early childhood. *Journal of Family violence, 18*(1), 43-57.

Straus, M. A., Hamby, S. L., Finkelhor, D., Moore, D. W., & Runyan, D. (1998). Identification of child maltreatment with the Parent–Child Conflict Tactics Scales: Development and psychometric data for a national sample of American parents. *Child Abuse & Neglect, 22*(4), 249-270. doi:10.1016/S0145-2134(97)00174-9
